# 4,4′-Diiodo-3,3′-dimethoxy­biphen­yl

**DOI:** 10.1107/S160053680801129X

**Published:** 2008-04-26

**Authors:** Qamar Ali, Muhammad Raza Shah, Donald VanDerveer

**Affiliations:** a HEJ Research Institute of Chemistry, International Center for Chemical and Biological Sciences, University of Karachi, Karachi 75270, Pakistan; b Molecular Structure Center, Chemistry Department, Clemson University, Clemson, SC 29634-0973, USA

## Abstract

The mol­ecules of the title compound, C_14_H_12_I_2_O_2_, lie on inversion centers and are linked by I⋯O inter­actions with inter­molecular distances of 3.324 (3) Å. The aromatic rings display no significant inter­calation or stacking inter­actions.

## Related literature

For related literature see: Sakai & Matile (2003 ▶); Sakai *et al.* (1997 ▶); Anelli *et al.* (2001 ▶); Baumeister *et al.* (2001 ▶); Fidzinski *et al.* (2003 ▶); Mullen & Wegner (1998 ▶); Schwab & Levin (1999 ▶); Sisson *et al.* (2006 ▶).
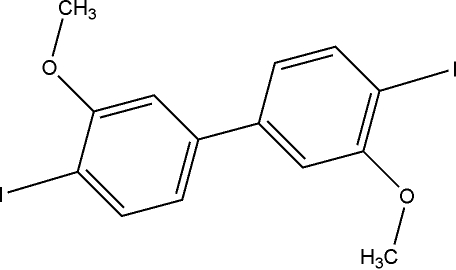


## Experimental

### 

#### Crystal data

C_14_H_12_I_2_O_2_
*M* *_r_* = 466.04
Monoclinic, 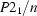

*a* = 6.8616 (14) Å
*b* = 7.7386 (15) Å
*c* = 13.435 (3) Å
β = 102.43 (3)°
*V* = 696.7 (2) Å^3^
*Z* = 2
Mo *K*α radiation
μ = 4.50 mm^−1^
*T* = 153 (2) K
0.36 × 0.26 × 0.24 mm

#### Data collection

Rigaku Mercury CCD diffractometer
Absorption correction: multi-scan (*REQAB*; Jacobson, 1998 ▶) *T*_min_ = 0.241, *T*_max_ = 0.337
5295 measured reflections
1417 independent reflections
1381 reflections with *I* > 2σ(*I*)
*R*_int_ = 0.039

#### Refinement

*R*[*F* ^2^ > 2σ(*F* ^2^)] = 0.024
*wR*(*F* ^2^) = 0.062
*S* = 1.13
1417 reflections
84 parameters
H-atom parameters constrained
Δρ_max_ = 1.08 e Å^−3^
Δρ_min_ = −0.90 e Å^−3^

### 

Data collection: *CrystalClear* (Rigaku/MSC, 2006 ▶); cell refinement: *CrystalClear*; data reduction: *REQAB* (Jacobson, 1998 ▶) and *CrystalClear*; program(s) used to solve structure: *SHELXTL* (Sheldrick, 2008 ▶); program(s) used to refine structure: *SHELXTL*; molecular graphics: *SHELXTL*; software used to prepare material for publication: *SHELXTL*.

## Supplementary Material

Crystal structure: contains datablocks I, global. DOI: 10.1107/S160053680801129X/pv2077sup1.cif

Structure factors: contains datablocks I. DOI: 10.1107/S160053680801129X/pv2077Isup2.hkl

Additional supplementary materials:  crystallographic information; 3D view; checkCIF report

## Figures and Tables

**Fig. 1.**
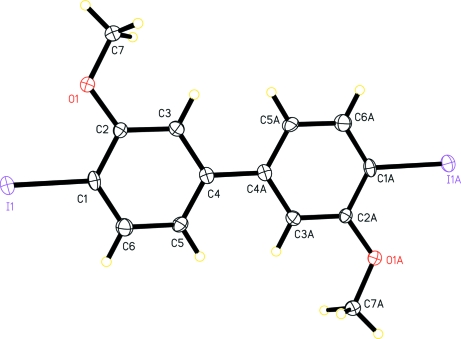
A thermal ellipsoid plot of the title compound drawn at 50% probability level. The symmetry related atoms have been identified by the letter A in atomic labels.

**Fig. 2.**
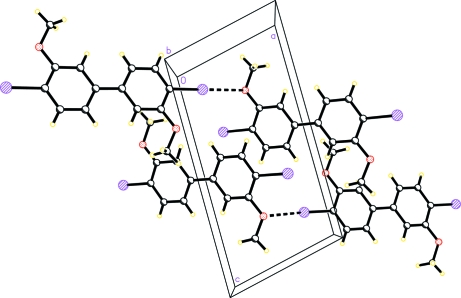
Packing diagram of the structure viewed down the b axis. Hydrogen bonds have been indicated with dashed lines.

## supplementary crystallographic information

### Comment

Over the last 25 years, much attention has been focused on the synthesis of artificial ion channels due to their potential applications in biomedical and material sciences (Schwab & Levin, 1999; Mullen & Wegner, 1998; Fidzinski *et al.*, 2003). The title compound has been used as a precursor for the synthesis of oligo(*p*-phenylene)s (Baumeister *et al.*, 2001) and as a source of hydrophobicity and rigidity (Sakai *et al.*, 1997; Sakai & Matile, 2003) in artificial ion channels. When a macrocycles like porphyrin is attached to the oligo(*p*-phenylene)s, the π-π stacking of porphyrin and the antiperiplanar arrangement of the oligo(*p*-phenylene)s should result in cylindrical self-assembly process and ultimately lead to the formation of functionalized pores (Sisson *et al.*, 2006). Furthermore, iodinated biphenyl has a bright prospect as X-ray contrast media (Anelli *et al.*, 2001).

In this paper, we report the sysnthesis and crystal structure of the title compound, (I). The molecules of (I) lie on crystallographic inversion centers. The I1—O1 intermolecular distance is 3.324 (3) Å which is significantly shorter than 3.50 Å, the sum of the van der Waals radii for I and O, supporting the idea that oxygen atom of methoxy disturbs the electronic cloud surrounding the iodide, hence creating polarization over iodide and subsequently causes reduction in the I1—O1 intermolecular distance. The crystal structures of three hexaiododerivatives of biphenyl have been reported (Anelli *et al.*, (2001).

### Experimental

Fast Blue B salt (*o*-dianisidine bisdiazotated zinc double salt, 10.00 g, 21 mmol), was added to a solution of KI (28 g, 0.17 mmol) in water (200 ml). The mixture was stirred at room temperature for 14 h. After dilution with dichloromethane, the crude reaction mixture was concentrated *in vacuo*. Purification of the crude product on silica gel (dichloromethane:hexane 1:4) followed by evaporation of the solvent under *in vacuo* gave the pure desired product as a pale yellow solid in 70% yield. Single crystals suitable for X-ray crystallography were obtained by slow evaporation of a solution of pale yellow solid in ethanol at room temperature.

### Refinement

All H atoms were geometrically positioned and were allowed to ride on the corresponding C-atoms with C—H = 0.96 Å and *U*_iso_(H) = 1.5*U*_eq_(C) of the attached C atom for methyl H atoms and 1.2*U*_eq_(C) for other H atoms. The highest peak in the final difference Fourier map corresponding to a residual electron density of 1.08 e/Å^3^ was located at 1.2 Å from H5 and was deemed meaningless.

### Crystal data

**Table:**

C_14_H_12_I_2_O_2_	*F*(000) = 436
*M**_r_* = 466.04	*D*_x_ = 2.222 Mg m^−^^3^
Monoclinic, *P*2_1_/*n*	Mo *K*α radiation, λ = 0.71073 Å
Hall symbol: -P 2yn	Cell parameters from 2340 reflections
*a* = 6.8616 (14) Å	θ = 3.0–26.4°
*b* = 7.7386 (15) Å	µ = 4.51 mm^−^^1^
*c* = 13.435 (3) Å	*T* = 153 K
β = 102.43 (3)°	Chip, colorless
*V* = 696.7 (2) Å^3^	0.36 × 0.26 × 0.24 mm
*Z* = 2	

### Data collection

**Table:**

Rigaku Mercury CCD diffractometer	1417 independent reflections
Radiation source: Sealed Tube	1381 reflections with *I* > 2σ(*I*)
Graphite Monochromator	*R*_int_ = 0.039
Detector resolution: 14.6306 pixels mm^-1^	θ_max_ = 26.4°, θ_min_ = 3.1°
ω scans	*h* = −8→8
Absorption correction: multi-scan (REQAB; Jacobson, 1998)	*k* = −9→9
*T*_min_ = 0.241, *T*_max_ = 0.337	*l* = −16→11
5295 measured reflections	

### Refinement

**Table:**

Refinement on *F*^2^	Secondary atom site location: difference Fourier map
Least-squares matrix: full	Hydrogen site location: inferred from neighbouring sites
*R*[*F*^2^ > 2σ(*F*^2^)] = 0.024	H-atom parameters constrained
*wR*(*F*^2^) = 0.062	*w* = 1/[σ^2^(*F*_o_^2^) + (0.0318*P*)^2^ + 1.081*P*] where *P* = (*F*_o_^2^ + 2*F*_c_^2^)/3
*S* = 1.13	(Δ/σ)_max_ < 0.001
1417 reflections	Δρ_max_ = 1.08 e Å^−^^3^
84 parameters	Δρ_min_ = −0.90 e Å^−^^3^
0 restraints	Extinction correction: *SHELXTL* (Sheldrick, 2008), Fc^*^=kFc[1+0.001xFc^2^λ^3^/sin(2θ)]^-1/4^
Primary atom site location: structure-invariant direct methods	Extinction coefficient: 0.0437 (19)

### Special details

**Table:**

Experimental. Refinement of F^2^ against ALL reflections. The weighted *R*-factor *wR* and goodness of fit S are based on F^2^, conventional *R*-factors *R* are based on F, with F set to zero for negative F^2^. The threshold expression of F^2^ > 2sigma(F^2^) is used only for calculating *R*-factors(gt) *etc*. and is not relevant to the choice of reflections for refinement. *R*-factors based on F^2^ are statistically about twice as large as those based on F, and R– factors based on ALL data will be even larger.
Geometry. All e.s.d.'s (except the e.s.d. in the dihedral angle between two l.s. planes) are estimated using the full covariance matrix. The cell e.s.d.'s are taken into account individually in the estimation of e.s.d.'s in distances, angles and torsion angles; correlations between e.s.d.'s in cell parameters are only used when they are defined by crystal symmetry. An approximate (isotropic) treatment of cell e.s.d.'s is used for estimating e.s.d.'s involving l.s. planes.

### Fractional atomic coordinates and isotropic or equivalent isotropic displacement parameters (Å^2^)

**Table:**

	*x*	*y*	*z*	*U*_iso_*/*U*_eq_	
I1	0.28040 (3)	0.14667 (2)	0.340534 (14)	0.01974 (14)	
O1	0.5702 (3)	0.3479 (3)	0.22987 (17)	0.0195 (5)	
C1	0.5402 (4)	0.2888 (3)	0.3985 (2)	0.0152 (5)	
C2	0.6482 (4)	0.3641 (3)	0.3325 (2)	0.0129 (6)	
C3	0.8273 (4)	0.4479 (4)	0.3731 (2)	0.0151 (5)	
H3	0.9008	0.5005	0.3278	0.018*	
C4	0.9026 (4)	0.4569 (3)	0.4787 (2)	0.0139 (5)	
C5	0.7896 (4)	0.3821 (4)	0.5427 (2)	0.0183 (6)	
H5	0.8366	0.3884	0.6153	0.022*	
C6	0.6107 (4)	0.2990 (4)	0.5026 (2)	0.0203 (6)	
H6	0.5353	0.2483	0.5475	0.024*	
C7	0.6900 (5)	0.4047 (5)	0.1613 (2)	0.0230 (7)	
H7A	0.7072	0.5277	0.1668	0.035*	
H7B	0.6251	0.3752	0.0927	0.035*	
H7C	0.8180	0.3493	0.1783	0.035*	

### Atomic displacement parameters (Å^2^)

**Table:**

	*U*^11^	*U*^22^	*U*^33^	*U*^12^	*U*^13^	*U*^23^
I1	0.01475 (17)	0.02317 (18)	0.02110 (17)	−0.00579 (6)	0.00339 (9)	−0.00124 (6)
O1	0.0194 (10)	0.0253 (12)	0.0136 (10)	−0.0088 (8)	0.0029 (8)	−0.0017 (7)
C1	0.0090 (11)	0.0127 (12)	0.0230 (13)	−0.0005 (10)	0.0019 (10)	−0.0010 (10)
C2	0.0140 (13)	0.0108 (13)	0.0137 (14)	0.0005 (9)	0.0024 (10)	−0.0009 (9)
C3	0.0156 (12)	0.0141 (12)	0.0161 (13)	−0.0002 (10)	0.0042 (10)	0.0009 (10)
C4	0.0119 (12)	0.0111 (11)	0.0180 (13)	0.0021 (10)	0.0019 (10)	0.0018 (10)
C5	0.0143 (13)	0.0267 (14)	0.0121 (13)	−0.0008 (12)	−0.0014 (10)	0.0032 (11)
C6	0.0148 (13)	0.0258 (15)	0.0200 (14)	0.0004 (12)	0.0028 (10)	0.0021 (12)
C7	0.0239 (14)	0.0306 (17)	0.0145 (14)	−0.0100 (14)	0.0038 (11)	−0.0008 (12)

### Geometric parameters (Å, °)

**Table:**

I1—C1	2.097 (3)	C4—C5	1.401 (4)
O1—C2	1.373 (4)	C4—C4^i^	1.493 (5)
O1—C7	1.429 (4)	C5—C6	1.388 (4)
C1—C6	1.380 (4)	C5—H5	0.9600
C1—C2	1.399 (4)	C6—H6	0.9600
C2—C3	1.392 (4)	C7—H7A	0.9599
C3—C4	1.404 (4)	C7—H7B	0.9599
C3—H3	0.9600	C7—H7C	0.9599
			
C2—O1—C7	117.8 (2)	C6—C5—C4	120.8 (3)
C6—C1—C2	120.0 (3)	C6—C5—H5	119.6
C6—C1—I1	119.4 (2)	C4—C5—H5	119.6
C2—C1—I1	120.5 (2)	C1—C6—C5	120.5 (3)
O1—C2—C3	123.8 (3)	C1—C6—H6	119.7
O1—C2—C1	117.0 (2)	C5—C6—H6	119.7
C3—C2—C1	119.3 (3)	O1—C7—H7A	109.5
C2—C3—C4	121.4 (3)	O1—C7—H7B	109.5
C2—C3—H3	119.3	H7A—C7—H7B	109.5
C4—C3—H3	119.3	O1—C7—H7C	109.5
C5—C4—C3	117.9 (2)	H7A—C7—H7C	109.5
C5—C4—C4^i^	121.2 (3)	H7B—C7—H7C	109.5
C3—C4—C4^i^	120.9 (3)		

Symmetry codes: (i) −*x*+2, −*y*+1, −*z*+1.
